# Low-intensity Pulsed Ultrasound regulates alveolar bone homeostasis in experimental Periodontitis by diminishing Oxidative Stress

**DOI:** 10.7150/thno.42508

**Published:** 2020-08-01

**Authors:** Siqi Ying, Minmin Tan, Ge Feng, Yunchun Kuang, Duanjing Chen, Jie Li, Jinlin Song

**Affiliations:** 1 College of Stomatology, Chongqing Medical University, Chongqing, China.; 2 Chongqing Key Laboratory of Oral Diseases and Biomedical Sciences, Chongqing, China.; 3 Chongqing Municipal Key Laboratory of Oral Biomedical Engineering of Higher Education, Chongqing, China.

**Keywords:** periodontitis, alveolar bone, oxidative stress

## Abstract

Periodontitis is a widespread oral disease that results in the loss of alveolar bone. Low-intensity pulsed ultrasound (LIPUS), which is a new therapeutic option, promotes alveolar bone regeneration in periodontal bone injury models. This study investigated the protective effect of LIPUS on oxidative stress in periodontitis and the mechanism underlying this process.

**Methods:** An experimental periodontitis model was induced by administering a ligature*.* Immunohistochemistry was performed to detect the expression levels of oxidative stress, osteogenic, and osteoclastogenic markers *in vivo*. Cell viability and osteogenic differentiation were analyzed using the Cell Counting Kit-8, alkaline phosphatase, and Alizarin Red staining assays. A reactive oxygen species assay kit, lipid peroxidation MDA assay kit, and western blotting were used to determine oxidative stress status *in vitro*. To verify the role of nuclear factor erythroid 2-related factor 2 (Nrf2), an oxidative regulator, during LIPUS treatment, the siRNA technique and Nrf2^-/-^ mice were used. The PI3K/Akt inhibitor LY294002 was utilized to identify the effects of the PI3K-Akt/Nrf2 signaling pathway.

**Results:** Alveolar bone resorption, which was experimentally induced by periodontitis* in vivo*, was alleviated by LIPUS via activation of Nrf2. Oxidative stress, induced via H_2_O_2_ treatment *in vitro*, inhibited cell viability and suppressed osteogenic differentiation. These effects were also alleviated by LIPUS treatment via Nrf2 activation. Nrf2 silencing blocked the antioxidant effect of LIPUS by diminishing heme oxygenase-1 expression. Nrf2^-/-^ mice were susceptible to ligature-induced periodontitis, and the protective effect of LIPUS on alveolar bone dysfunction was weaker in these mice. Activation of Nrf2 by LIPUS was accompanied by activation of the PI3K/Akt pathway. The oxidative defense function of LIPUS was inhibited by exposure to LY294002 *in vitro*.

**Conclusions:** These results demonstrated that LIPUS regulates alveolar bone homeostasis in periodontitis by attenuating oxidative stress via the regulation of PI3K-Akt/Nrf2 signaling. Thus, Nrf2 plays a pivotal role in the protective effect exerted by LIPUS against ligature-induced experimental periodontitis.

## Introduction

Periodontitis is a widespread oral disease that affects almost half of the adult population worldwide 1. Its initiation and progression is considered to be due to an imbalance between inflammatory and host immune responses caused by periodontal bacteria 2. Treatments currently available for periodontitis include mechanical debridement, anti-inflammatory medication and regenerative surgery 3. However, due to unsatisfactory outcomes associated with the use of anti-inflammatory drugs, alternative supplemental therapies are being increasingly considered as treatments for periodontitis.

The therapeutic potential of physical stimulation as an effective treatment for periodontitis has been described 4, 5. Low-intensity pulsed ultrasound (LIPUS) is a non-invasive ultrasound technique that transmits low frequency and low-intensity sound waves into tissues as acoustic pressure waves 6, 7. Conversion of mechanical signals emanating from these pressure waves to biochemical responses results in downstream effects 8. Multiple experiments have demonstrated that LIPUS modulates bone tissue regeneration and accelerates bone repair processes by upregulating bone specific genes and downregulating osteoclast differentiation 9, 10. Our latest studies indicated that LIPUS promoted alveolar bone regeneration *in vivo* 11 and facilitated osteogenic differentiation *in vitro* 12. However, the precise mechanism by which LIPUS exerts protective effects on periodontitis remains unclear, thereby limiting the clinical application of LIPUS.

Oxidative stress is defined as an imbalance between the excessive production of reactive oxygen species (ROS) and a relative deficiency of antioxidants 13. Numerous studies have demonstrated that oxidative stress is one of the pathophysiological mechanisms underlying inflammation, as well as periodontitis 14-18. It was further established that periodontitis may be initiated by increased oxidative damage 19. A previous study conducted by us revealed that oxidative stress impedes osteogenic differentiation and decreases regenerative potential 20. Therefore, it is evident that oxidative stress plays an essential role in the pathological process of periodontitis. Besides, exploring modalities that decrease oxidative stress may reveal new avenues that show potential as periodontal treatments. Nuclear factor erythroid 2-related factor 2 (Nrf2) is a redox-sensitive transcription factor that upregulates a battery of antioxidant genes that defend against oxidative stress 21. It has been reported that Nrf2 deficiency may accelerate osteoclastogenesis and elevate the oxidative damage 22. Nrf2^-/-^ mice exhibited more bone loss compared to wild-type mice 23. By contrast, activated Nrf2 protected periodontal tissue from oxidative stress, periodontitis, and alveolar bone destruction 24-26. Most importantly, LIPUS induced cytoprotective effects by reducing oxidative stress 26. Therefore, we hypothesized that LIPUS may be useful as a new supplemental therapy for periodontitis.

The objectives of the present study were to elucidate the mechanisms underlying LIPUS-mediated protection against periodontitis and to determine the association between the roles played by LIPUS, oxidative stress, and Nrf2 in the development of periodontitis-induced alveolar bone destruction.

## Materials and Methods

### LIPUS application instructions

The LIPUS exposure device, manufactured by National Engineering Research Center of Ultrasound Medicine (Chongqing Medical University, Chongqing, China), consisted of an array of 6 transducers (each with a diameter of 34.8 mm), which were specifically designed for use in a 6-well culture plate. The LIPUS exposure device contains a focused transducer, which was positioned at the maxillary first and second molars to direct the acoustic beam to the desired region.

The parameters of LIPUS used in the present study consisted of a 1.5 MHz ultrasound signal with a 200 ms burst sine wave, pulsed 1:4 (2 ms on and 8 ms off) with a repetition rate of 1.0 kHz, and an average spatial and temporal intensity of 30, 60, and 90 mW/cm^2^. The percentage permeability of ultrasound through the ultrasonic coupling gel, as determined via an ultrasound power meter (UPM-DT-1 AV; Ohmic Instruments, Easton, MD, USA), was 63.56±2.02. Control groups were handled in the same way as treatment groups, except for the fact that the ultrasound generator was not switched on.

### Animal and treatment schedule

#### Animals

All experiments in this study were conducted in accordance with the Declaration of Helsinki, ARRIVE guidelines, and National Institutes of Health Guide for the Care and Use of Laboratory Animals under protocols reviewed and approved by the Ethics Committee of the College of Stomatology, Chongqing Medical University. C57BL/6 mice and Sprague-Dawley (SD) rats were purchased from the Experimental Animal Center of Chongqing Medical University (Chongqing, China), while Nrf2^-/-^ mice with a C57BL/6 background were donated by Professor Gangyi Yang at the second affiliated hospital of Chongqing Medical University. All animals were housed in a specific pathogen-free room at Chongqing Key Laboratory of Oral Diseases and Biomedical Sciences and received water and food *ad libitum* from the Animal Care Facility Service.

#### Group allocation

Twenty SD rats were randomly divided into 4 groups of 5 each. The study groups were as follows: Control (no treatment), Ligature (ligature-induced experimental periodontitis for 14 days), LIPUS-only (LIPUS treatment for 14 days) and Ligature + LIPUS (experimental periodontitis before LIPUS treatment for 14 days). In a separate study, Nrf2^-/-^ and Wild-type (WT) C57BL/6 mice were divided into 5 groups of 5 each. The study groups were as follows: Nrf2^+/+^ (WT with no treatment), Nrf2^-/-^ Control (Nrf2^-/-^ with no treatment), Nrf2^-/-^ Ligature (Nrf2^-/-^ with ligature-induced experimental periodontitis for 8 days), Nrf2^-/-^ LIPUS-only (Nrf2^-/-^ with LIPUS treatment for 8 days), Nrf2^-/-^ Ligature + LIPUS (Nrf2^-/-^ with experimental periodontitis and LIPUS treatment for 8 days).

**Figure 1:**
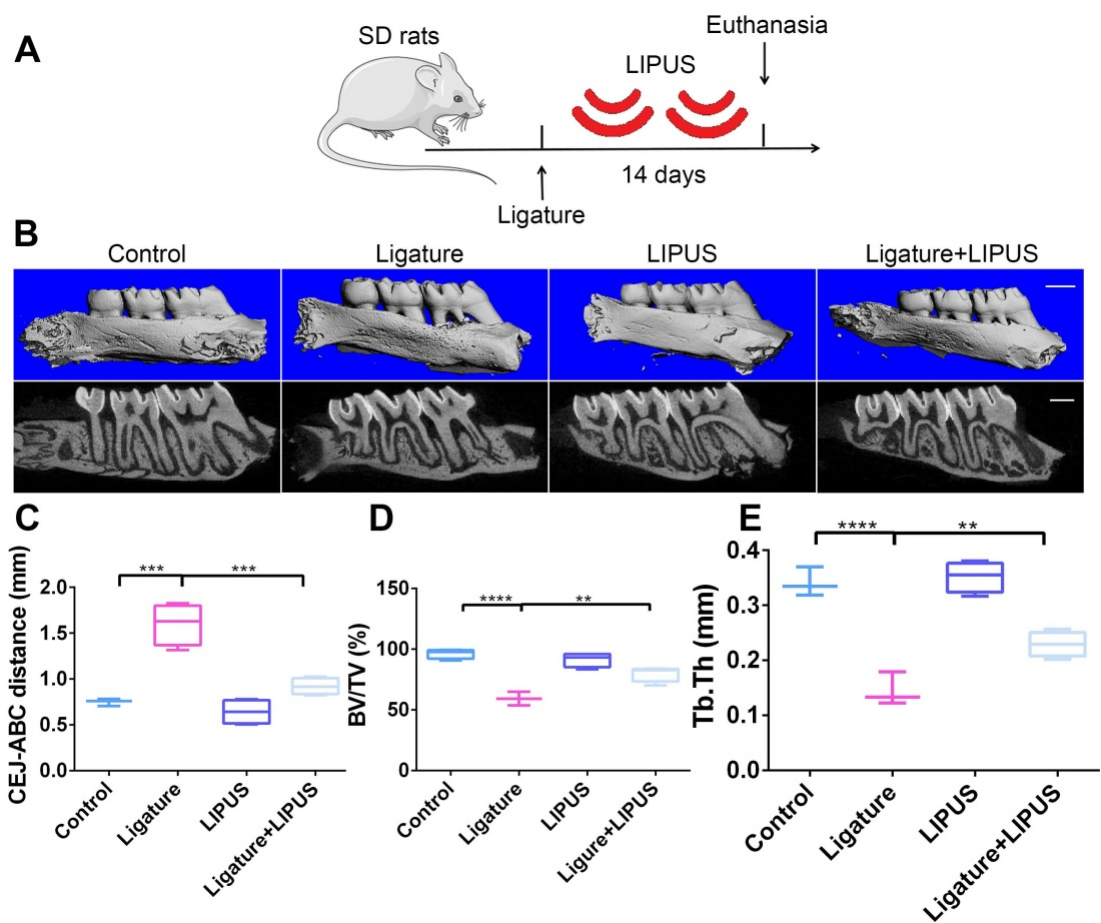
** LIPUS alleviates ligature-induced alveolar bone destruction in periodontitis.** (**A**) SD rats were subjected to subcutaneous ligature insertion for 14 d, with or without LIPUS treatment (30 min/day, with 200 ms pulses and 1.5 MHz) for 14 d. (**B**) Micro-CT image of the alveolar bone (Scale bar = 1 mm), (**C**) CEJ-ABC distance, (**D**) BV/TV, and (**E**) Tb.Th. Control (no treatment), Ligature (ligature-induced experimental periodontitis), LIPUS (only LIPUS treatment), Ligature + LIPUS (experimental periodontitis before LIPUS treatment). Data are presented as the mean ± SEM (n = 3). *, *p* < 0.05; **, *p* < 0.005; ***, *p* < 0.0005; ****, *p* < 0.00005.

Each animal in the LIPUS-only group was treated with LIPUS daily for 30 min. In the ligature group and ligature + LIPUS group, animals underwent insertion of the ligature. In the latter group only, animals were also treated with LIPUS daily for 30 min after insertion of the ligature, whereas control animals were handled identically but without the LIPUS stimulation (Figure 1A and Figure 8B).

#### Animal ligature-induced periodontitis model

Establishing and inducing the experimental periodontitis model was performed as previously described 27. Briefly, a 5-0 silk ligature was inserted between the maxillary left first and second molars and knots were tied on both ends to secure the ligature. The ligatures were examined daily to ensure that they remained in place during the experimental period. Periodontal inflammation and bone loss in this model is initiated by massive local accumulation of bacteria on the ligature-treated molars 28. The contralateral side of each mouse was left without ligature treatment. A second set of controls included mice that were not treated with ligatures on either side.

#### Micro-computed tomography

The upper jaws were dissected and scanned using a high-resolution CT system (Skyscan 1172; Skyscan, Aartselaar, Belgium). Three-dimensional reconstructions were generated using Amira software. Scans were performed using the following scanner settings: X-ray source voltage 70 kVp, current 114 µA, and power 8 W. CT scan (Scanco Medical AG) settings were high resolution, voxel size of 15 µm, slice thickness of 0.01 mm, and FOV/diameter of 30.7 mm, while an integration time of 250 ms micro-CT was used to assess the loss of alveolar bone. To qualify bone loss, the six-site total cementoenamel junction and alveolar bone crest (CEJ-ABC) distance for the ligature-treated side of each mouse was subtracted from the six-site total CEJ-ABC distance of the contralateral side (no ligature treatment) of the same mouse. Bone density measurements were taken in samples oriented using the Scanco Medical analysis software. The maxillae were oriented with the first molar CEJ parallel to each other in the sagittal and coronal planes. The crowns of the first, second, and third molars were visible in the axial plane. After orientation, a 40-slice volume set at a threshold of 75 was considered to be the region of interest for analysis. Analysis started at the slice in which furcation first appeared, proceeding apically. BV/TV percentage and Tb.Th. values were recorded and averaged for each group (n ≥ 3/group for all micro-CT analyses).

#### Paraffin sections

Following micro-CT scans, specimens were decalcified in 10% EDTA for at least 1 month, and then blocked parallel to the long axis of the left first and second molar on the lingual margin in order to allow subsequent histologic sections to show the interproximal region between the two teeth. Specimens were then dehydrated and embedded in paraffin, and 4 μm sections were made.

#### Immunohistochemistry

Periodontal sections were blocked in 10% normal goat serum; incubated overnight with primary antibodies against 3-nitrotyrosine (3-NT) (1:200) (Abcam, Cambridge, UK), 8-Hydroxy-2'-deoxyguanosine 8-OHdG (1:200) (Abcam), Nrf2 (1:300) (Abcam), and HO-1 (1:300) (Abcam), Interleukin (IL)-6 (1:200) (Bioss), tumor necrosis factor alpha TNF-α (1:100) (Bioss), receptor activator of NF-ҡB ligand RANKL (1:300) (Abcam), alkalinephosphatase ALP (1:300) (Abcam) and osteocalcin OCN (1:200) (Abcam) at 4 °C; and stained with HRP-conjugated secondary antibodies (ZSJQ-BIO, Beijing, China). Diaminobenzidine (DAB) was used as a substrate for color development. All slides were counterstained with hematoxylin. Images of histologically stained sections were obtained using a BX41 microscope (Olympus, Japan). The H-score method 29 was applied for calculating the staining score of each sample, which was to multiply the immunoreaction intensity (negative: 0, weak: 1, moderate: 2, strong: 3) by the staining extent score (0%-100%). According to the H-score, the stained samples were divided into four groups: negative (-; 0), weak (+; 0~1), moderate (++; 1~1.5), and strong (+++; 1.5~3). Samples with a negative or weak H-score were determined to be the low protein expression group, whereas those with a moderate or strong H-score were classified as the high protein expression group.

#### TRAP staining

The TRAP stain was conducted with a Leukocyte Acid Phosphatase kit (Nanjin jianchen Bioengineering Institute, Nanjing, China). The TRAP-positive cells were photographed and counted with the Olympus BX41 microscope.

#### MPO flow analysis

Gingival tissues were homogenized in a solution composed of 3% type I collagenase (Sigma-Aldrich, St. Louis, MO, USA) in 50 mM sodium phosphate buffer. After homogenization, cells were fixed, permeabilized, and stained intracellularly with Anti-Myeloperoxidase antibody (Abcam) for 60 min at 4 °C, and then incubated with Goat Anti-rabbit IgG/Alexa Fluor 488 antibody (Bioss, Beijing, China) for 60 min. At least 1 × 10^4^ cells were analyzed per sample. Flow cytometry was performed on a BD Influx flow cytometer (BD Biosciences, San Jose, CA).

### Cell and treatment schedule

#### Cell culture

Healthy volunteers aged from 10 to 20 years, with informed consent from guardians, donated their teeth for orthodontic extraction treatment. Isolation and culturing of periodontal ligament cells (PDLCs) were performed as previously described 23. Briefly, periodontal ligament tissue was carefully scraped from the middle third of the root and digested with 3 mg/mL of type I collagenase (Sigma-Aldrich) dissolved in phosphate-buffered saline at 37 °C for 30 min. Following full digestion, the periodontal ligament tissue suspension was seeded in α-Minimum Essential Medium (α-MEM; Hyclone, Logan, UT, USA) supplemented with 10% fetal bovine serum (FBS; Gibco, USA), 100 U/mL of penicillin and 100 µg/mL of streptomycin. The culture was incubated at 37 °C in a humidified atmosphere with 95% air and 5% CO_2_. The cell culture medium was changed every 3 days. PDLCs were passaged at a ratio of 1:3 when they reached approximately 80% confluence. Pooled cell samples from the 3rd passage were used for further treatment.

#### Cell treatment

In order to establish the cellular oxidative stress model, PDLCs were exposed to H_2_O_2_ (Sigma) for 6 h after reaching approximately 90% confluence and further incubated in culture medium for 8 h. PDLCs were pretreated with LIPUS 30 min prior to the addition of H_2_O_2_ to investigate its effect on oxidative stress. All experiments were performed in triplicate.

#### Cell viability and evaluation of cell proliferation

PDLC proliferation was evaluated using Cell Counting Kit-8 (Beyotime Biotechnology, Shanghai, China). The treated cell suspension (5 × 10^3^ cells/well) was seeded on a 96-well plate for 48 h. Next, cells were treated with CCK-8 reagent (Beyotime) and incubated for 2 h at 37 °C. Absorbance at 450 nm was measured using a microplate reader (Perkin Elmer, USA). All experiments were repeated in triplicate.

### Osteogenic differentiation of PDLCs

Osteogenic induction was applied to detect the osteogenic differentiation capacity of PDLCs induced by H_2_O_2_ in the presence or absence of LIPUS pretreatment. Cells were seeded at a density of 1 × 10^5^ cells per well in a 6-well culture plate and incubated. When the cells reached 80% confluency, the medium was replaced with stem osteogenic differentiation medium [a-MEM supplemented with 10% FBS, 50 µg/mL of ascorbic acid (Sigma), 5 mM L-glycerophosphate (Sigma), and 100 nM dexamethasone (Sigma) for 7-21 days depending on the experiment.

Alkaline phosphatase (ALP) staining was determined using NBT/BCIP (Beyotime) according to the manufacturer's protocol after 7 days of osteogenic induction. ALP activity was evaluated using an AKP analysis kit (Nanjing Jiancheng Bioengineering Institute, China) after 7 days, according to the manufacturer's instructions.

Calcium accumulation was detected by fixing with 4% fixative solution (Solarbio Co., Ltd., Beijing, China) and staining with 0.2% Alizarin Red (Solarbio) after 21 days of osteogenic induction. To quantify mineralization, cells stained with Alizarin Red were destained with 10% cetylpyridinium chloride monohydrate (Solarbio), following which the extracted stain was transferred to a 96-well plate, and the absorbance at 405 nm was measured using a microplate reader (Perkin Elmer, USA).

### Detection of oxidative stress

Intracellular ROS were detected using an ROS Assay Kit (Beyotime, China). Following H_2_O_2_ exposure for 6 h, cells in six-well plates were incubated with 1 mL of 0.1% DCFH-DA diluted in a-MEM at 37 °C for 20 min and washed thrice with a-MEM. Green fluorescence was examined under a fluorescence microscope. Three days following H_2_O_2_ exposure, PDLCs were collected and protein was extracted. Protein concentration was determined using a BCA protein assay kit (Beyotime). MDA activity was detected using a Lipid Peroxidation MDA Assay Kit (Beyotime) according to the manufacturer's protocol. MDA concentrations were expressed as mol/g protein.

### Quantitative RT-PCR analysis

Total RNA was extracted from PDLCs in a six-well plate using RNAiso Plus (Takara, Beijing, China). Next, 500 ng of total RNA was used as the template to synthesize cDNA using 5 X PrimeScript RT Master Mix (Takara). For PCR amplification, a 10-µL reaction volume was used, comprising 5 µg of 2 X TB Green Fast qPCR Mix (Takara), 0.1 mM of each primer, 1 µL of fivefold diluted cDNA and diethyl pyrocarbonate water (Sangon Biotech). The reaction and detection were conducted using a CFX96 Real-Time PCR Detection System (Bio-Rad Laboratories, Inc., Hercules, CA, USA). The cycle threshold (Ct) values were collected and normalized to the level of glyceraldehyde-3-phosphate dehydrogenase (*GAPDH*), a housekeeping gene. The primer sequences used for runt-related transcription factor-2* (Runx2)*, *ALP, Nrf2, HO-1,* and *GAPDH* were as follows: *ALP* (F) 5′-TAAGGACATCGCCTACCAGCTC-3′, (R) 5′-TCTTCCAGGTGTCAACGAGGT-3′, *OCN* (F) 5′-GAATAGACTCCGGCGCTACC-3′, (R) 5′-AGCTCGTCACAATTGGGGTT-3′, *RANKL* (F) 5′-AGGATGCAACATACTTTGGG-3′, (R) 5′-TTGGGGCCATGCCTCTTAGT-3′, *IL-6* (F)5′-5GCCCTTCAGGAACAGCTATG-3′, (R) 5′-CAGAATTGCCATTGCACAAC-3′, *TNF-α* (F)5′-TGATCGGTCCCAACAAGGA-3′, (R) 5′-TGCTTGGTGGTTTGCTACGA-3′, *Nrf2* (F) 5′-TCAGAAACCAGTGGATCTGCC-3′, (R) 5′-AAGTGACTGAAACGTAGCCGA-3′, *HO-1* (F) 5′-CTCCCAGGGCCATGAACTTT-3′, (R) 50-GGGAAGATGCCATAGGCTCC-3′, and *GAPDH* (F) 50-CTTTGGTATCGTGGAAGGACTC-3′, (R) 5′-GTAGAGGCAGGGATGATGTTCT-3′.

### Western blotting analysis

The total protein content of the cells was isolated using RIPA lysis buffer (Beyotime) with 1 mM phenylmethanesulfonyl fluoride (PMSF; Beyotime), and protein concentration was measured using a BCA protein assay kit (Beyotime). For each protein sample, 30 µg was separated via sodium dodecyl sulfate-polyacrylamide gel electrophoresis (SDS-PAGE; Beyotime), followed by transfer to a polyvinylidene difluoride membrane (PVDF; Bio-Rad). After blocking with 5% nonfat milk, the membranes were incubated with primary antibodies against 3-NT (1:2000) (Abcam), 4-Hydroxynonenal (4-HNE) (1:500) (Abcam), Phospho-Nrf2 (1:5000) (Abcam), Nrf2 (1:1500) (Abcam), HO-1 (1:2000) (Abcam), Phospho-Akt (1:1000) (Cell Signaling Technology, MA, USA), Akt (1:1500) (Cell Signaling Technology), and GAPDH (1:1000) (ZenBio, Chengdu, China) overnight at 4 °C, followed by the respective secondary antibodies: horseradish peroxidase (HRP)-conjugated goat anti-rabbit IgG (1:3000) (ZenBio) or HRP-conjugated goat anti-mouse IgG (1:3000) (ZenBio). Bands were detected using electrochemiluminescence plus reagent (Bio-Rad, USA). Finally, the intensity of these bands was quantified using ImageJ software (Version 1.8.0; National Institutes of Health, USA).

### RNA silencing

In order to knock down Nrf2 expression, 1 × 10^5^ PDLCs were transfected with siRNA-Nrf2 and siRNA-Negative control (siRNA-NC), purchased from Sangon (Shanghai, China). Transient siRNA transfection was performed with Lipohigh reagent (Sangon) according to the manufacturer's instructions. Following 24 h of transfection, the cells were treated with LIPUS and H_2_O_2_. Transfection efficiency was confirmed via qPCR and western blotting.

### Immunofluorescence

Cells were treated with LIPUS and H_2_O_2_ to induce the translocation of Nrf2 into the nucleus. Meanwhile, PDLCs were pretreated with LY294002, a PI3K/Akt inhibitor. Following 48 h of treatment, cells were fixed using 4% paraformaldehyde and blocked with goat serum for 1 h. Cells were then incubated overnight with Nrf2 antibodies (1:500) (Abcam), and subsequently with goat anti-rabbit antibodies (1:400) (Bioss, China) conjugated with Alexa Fluor 555 for 1 h, on the following day. Cells were stained with DAPI. Images were acquired using an Olympus microscope (Olympus).

### Statistical analysis

An unpaired Student's *t*-test was used when two independent groups were analyzed. For multiple comparisons, analysis of variance (ANOVA) was performed (one-way or two-way ANOVA) with a Tukey's post hoc test. Data are expressed as mean ± standard error of the mean (SEM). *p*-values of <0.05 were considered statistically significant. Analyses were performed using GraphPad Prism software (version 6.0, USA).

## Results

### LIPUS alleviates ligature-induced alveolar bone destruction in periodontitis

To determine whether LIPUS protects against ligature-induced experimental periodontitis, rats were treated with or without LIPUS daily for 14 days (Figure 1A). The CEJ-ABC distance reflects the degree of bone resorption quantitatively. BV/TV and Tb.Th. were used for bone volume density. Bone resorption in the ligature group was increased compared with that of the control group. However, it was alleviated by LIPUS. There was no significant difference in alveolar bone destruction between the LIPUS-only group and the control group (Figure 1B-C). In addition, the ligature group showed a significant decrease in BV/TV and Tb.Th. compared with those of the controls, whereas LIPUS treatment significantly increased the levels of BV/TV and Tb.Th. Additionally, no difference was observed in the LIPUS treatment only compared with the control (Figure 1D-E).

### LIPUS alleviates ligature-induced oxidative stress via Nrf2 Pathway

**Figure 2:**
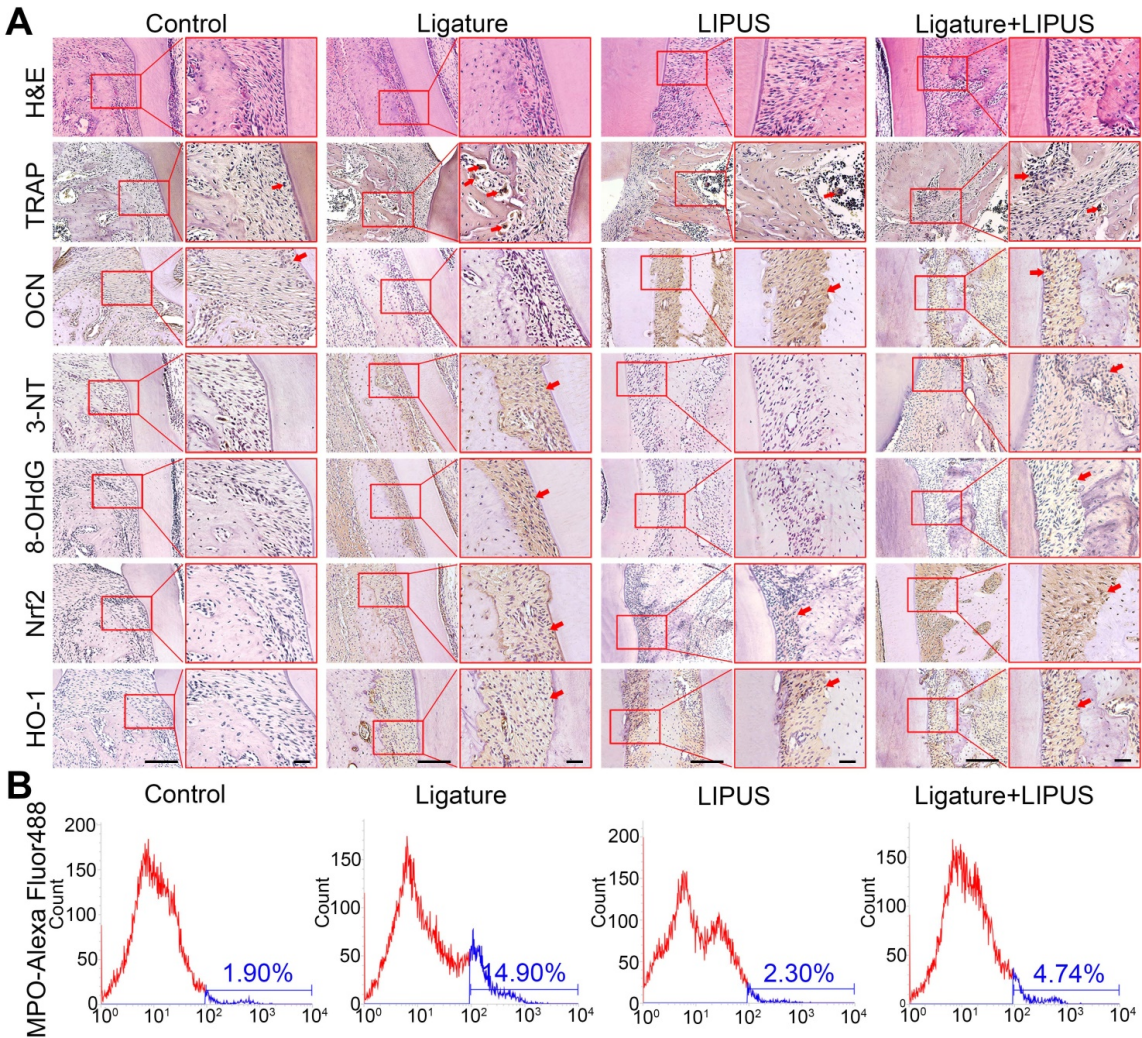
** LIPUS alleviates ligature-induced oxidative stress via Nrf2 Pathway.** (**A**) H&E staining at 200× and 400× magnification. TRAP staining, immunohistochemical staining of OCN, 3-NT, 8-OHdG, Nrf2, HO-1 at 200× and 400× (red box) magnification (Scale bar = 100 µm), (**B**) MPO flow analysis. Red arrows indicate positive staining.

**Figure 3:**
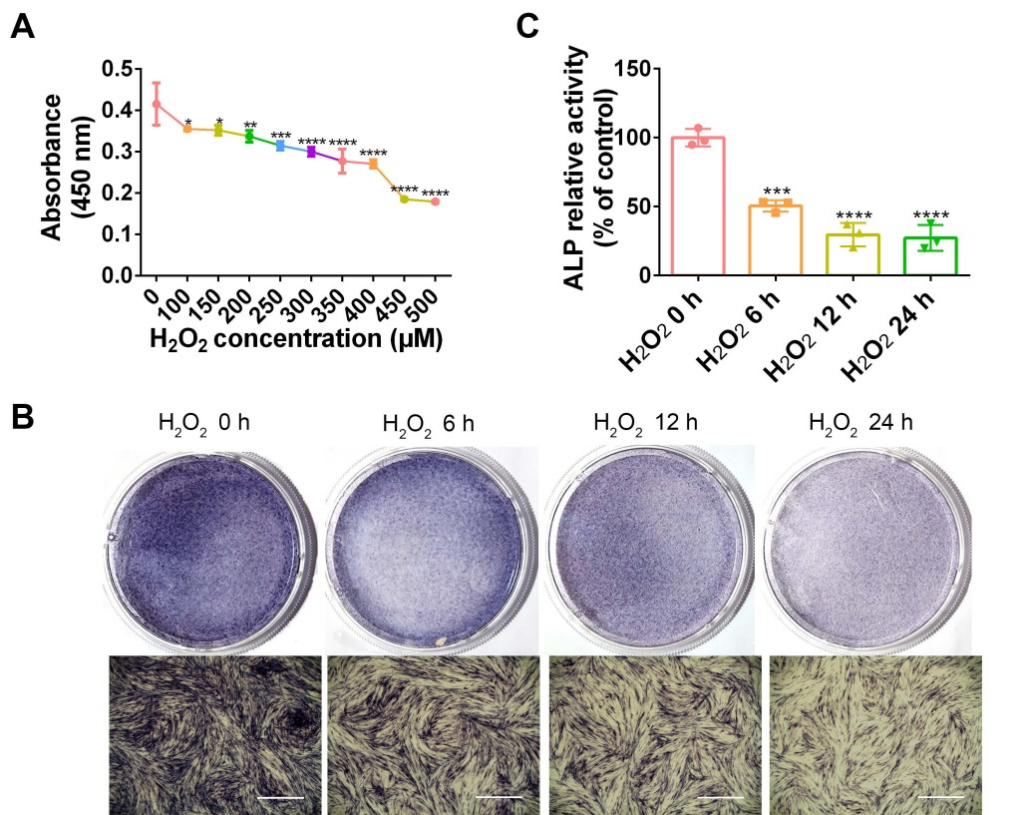
** H2O2 inhibits cell viability and osteogenic differentiation.** (**A**) Cell viability detected by the CCK-8 assay at different concentration of H2O2. Osteogenic differentiation at different exposure periods for H2O2 detected by ALP staining (Scale bar = 1 mm) (**B**) and ALP activity assay (**C**). Data are presented as the mean ± SEM (n = 3). *, *p* < 0.05; **, *p* < 0.005; ***, *p* < 0.0005; ****, *p* < 0.00005.

Histochemical analyses of decalcified H&E-stained bone sections indicate the protective effect exerted by LIPUS on ligature-induced experimental periodontitis models (Figure 2A). To clarified osteoblast and osteoclast activity, we stained ALP and OCN as osteogenic markers as well as IL-6, TNF-α, RANKL, and TRAP^+^ cells as osteoclastogenesis molecular markers. In the immunohistochemistry evaluation, ALP and OCN levels were significantly alleviated in the ligature group as compared to controls, with a marked increase in osteoblast activation after LIPUS treatment (Figure 2A, Figure S1 and Figure 3A). RANKL, IL-6, TNF-α, and TRAP staining was the complete opposite (Figure 2A and S1). This further corroborates the results by showing that LIPUS reduced alveolar bone destruction in experimental periodontitis models. To investigate whether oxidative stress played a role in ligature-induced experimental periodontitis and LIPUS protection, we evaluated oxidative stress biomarkers, such as 3-NT, 8-OHdG, and MPO. Immunohistochemistry staining showed that rats in the ligature group showed significantly elevated levels of 3-NT and 8-OHdG-positive cells, compared with those in the control, while the 3-NT and 8-OHdG levels were reduced following LIPUS treatment (Figure 2A and Figure S3B). Flow cytometry analysis revealed significantly increased MPO expression in ligature groups, and levels of MPO expression descended significantly in ligature + LIPUS groups (Figure 2B). However, no significant difference was observed between the levels of these biomarkers in the control and LIPUS groups. Furthermore, Nrf2 and HO-1 expression levels in each group were examined, and the results showed that ligatures induced an increase in the levels of Nrf2 and HO-1, whereas treatment with LIPUS further significantly increased the levels of Nrf2 and HO-1 expression (Figure 2A and Figure S3B).

### H_2_O_2_ inhibits PDLC viability and osteogenesis

As a common ROS inducer, H_2_O_2_ was used in the following study to mimic oxidative stress in periodontitis. To determine a dosage of H_2_O_2_ that would be adequate to establish the cellular oxidative stress model, PDLCs were stimulated with diverse concentrations of H_2_O_2_, followed by measurement of cell viability. Compared with non-treated cells, cell viability was significantly reduced by 100 and 150 µM H_2_O_2_ (*p* < 0.05), 200 µM H_2_O_2_ (*p* < 0.01), and 250 µM H_2_O_2_ (*p* < 0.005). Accordingly, the H_2_O_2_ concentration that would be most suitable for use in subsequent experiments was ascertained to be 200 µM (Figure 3A). ALP staining in H_2_O_2_-exposed PDLCs was significantly decreased compared to staining at 0 h, in a time dependent manner (Figure 3B-C). The results suggest that induction by H_2_O_2_ for 6 h was adequate and this was applied in the subsequent studies.

### LIPUS mitigates H_2_O_2_-induced inhibited cell viability and suppresses osteogenesis

**Figure 4:**
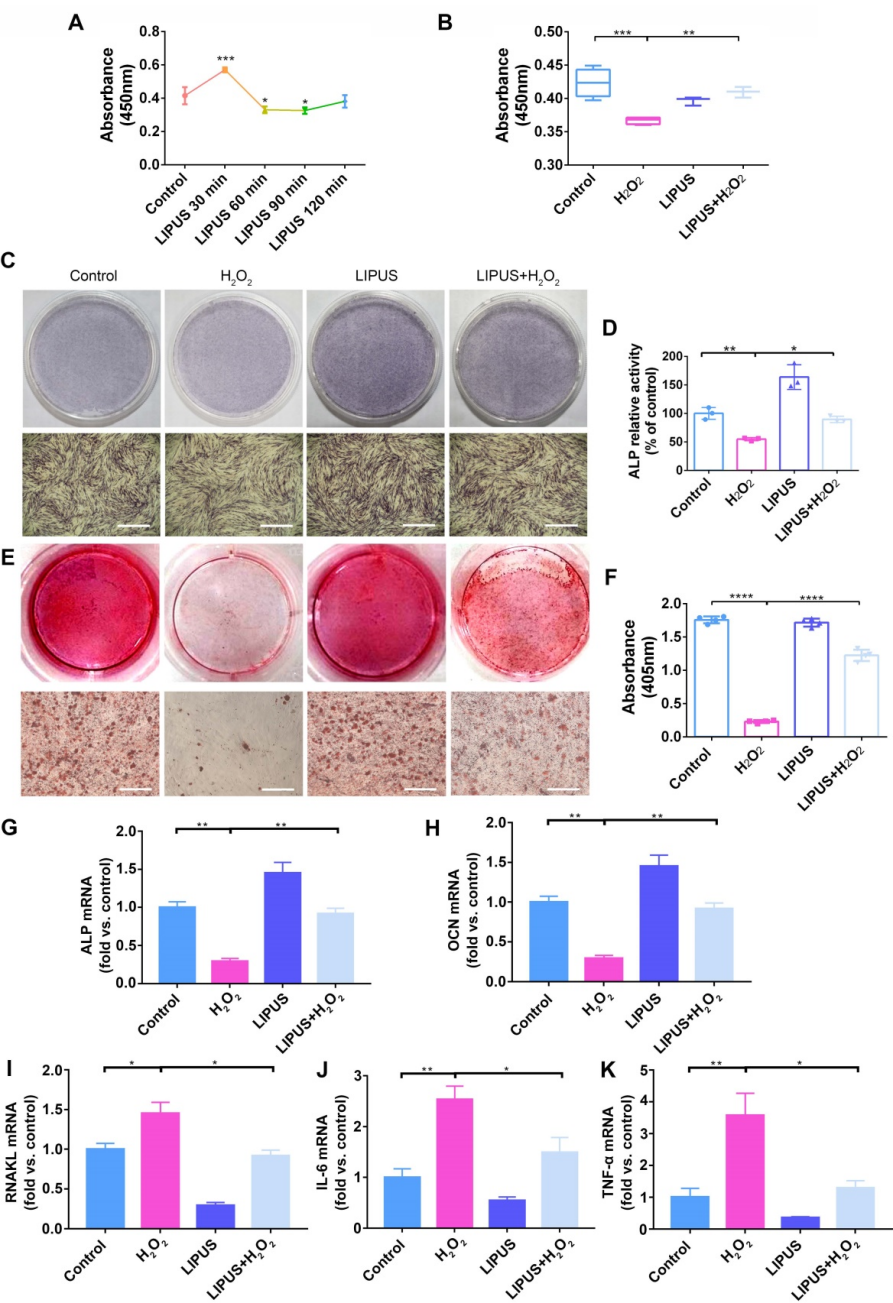
** LIPUS mitigates H2O2-induced inhibited cell viability and suppressed osteogenesis.** (**A**) Cell viability detected by the CCK-8 assay at different treatment periods for LIPUS. (**B**) Cell viability detected by the CCK-8 assay under H_2_O_2_ exposure and LIPUS treatment. Osteogenic differentiation detected by (**C**) ALP staining (Scale bar = 1 mm), (**D**) ALP activity, (**E**) Alizarin Red staining (Scale bar = 1 mm), (**F**) quantification of ARS staining, and gene expression of (**G**) *ALP*, (**H**) *OCN*, (**I**) *RANKL*, (**J**) *IL-6*, and (**K**) *TNF-α*. Data are presented as the mean ± SEM (n = 3). *, *p* < 0.05; **, *p* < 0.005; ***, *p* < 0.0005; ****, *p* < 0.00005.

The viability of PDLCs was measured in order to determine the effects of LIPUS on H_2_O_2_-induced suppression of cell viability and osteogenesis. A 30 min/day stimulation with LIPUS significantly enhanced cell viability compared with that of the other groups (Figure 4A). Simultaneously, cell viability, which was markedly lower than that of the control following H_2_O_2_-induction, was reversed by LIPUS treatment (Figure 4B). Therefore, the above results suggest that LIPUS rescued cell viability and promoted proliferation.

Next, to investigate the effects of LIPUS on both early and late stages of osteogenic differentiation in PDLCs, ALP and calcium were respectively evaluated. ALP and ARS (for calcium detection) staining results indicated that ALP and ARS in H_2_O_2_-exposed PDLCs, which were stained less compared to those of the control, were higher following LIPUS treatment (Figure 4C-E). We consistently observed that activity of ALP and calcium content in H_2_O_2_-exposed PDLCs were lower compared with those of the control, and that the levels of ALP activity and calcium content were attenuated by LIPUS treatment (Figure 4D-F). These data suggest that H_2_O_2_ inhibited both early as well as late stages of osteogenic differentiation in PDLCs, while LIPUS rescued the inhibitory effects of H_2_O_2_. Similarly, osteogenic differentiation and osteoclast differentiation were further evaluated via quantification of mRNA expression of the associated marker genes, *OCN*, *ALP*, *RANKL*, *IL-6*, and *TNF-α*. Both *OCN* and *ALP* were significantly downregulated following H_2_O_2_ induction, but upregulated following treatment with LIPUS (Figure 4G-H). *RANKL, IL-6*, and *TNF-α* stimulated by H_2_O_2_ were higher than those in the control, while those enhancements were inhibited by LIPUS treatment (Figure 4I-K).

### LIPUS mitigates H_2_O_2_-induced oxidative stress

**Figure 5:**
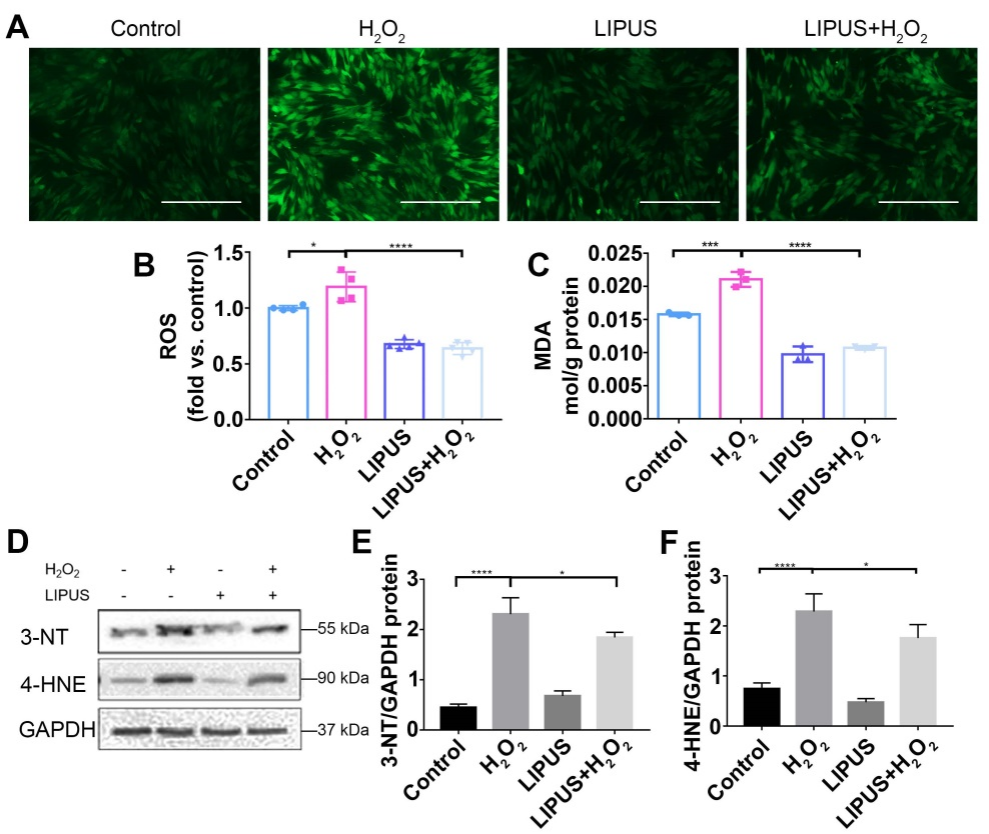
** LIPUS mitigates H2O2-induced oxidative stress.** Intracellular ROS measured by (**A**) DCFH-DA staining (Scale bar = 400 µm) and (**B**) DCFH-DA fluorescence intensity. (**C**) The MDA content of PDLCs was measured by MDA assay kit. Expression of 3-NT and 4-HNE were evaluated by (**D**) western blot. The representative expression of (**E**) 3-NT and (**F**) 4-HNE were measured by semi-quantitative analysis. Data are presented as the mean ± SEM (n = 3). *, *p* < 0.05; **, *p* < 0.005; ***, *p* < 0.0005; ****, *p* < 0.00005.

Oxidative stress has been reported to play a negative role in the progression of periodontitis 30. To investigate whether LIPUS protects PDLCs against H_2_O_2_-induced oxidative stress, we performed an ROS activity assay and an MDA assay. The results showed that ROS accumulated in PDLCs after 6 h of treatment with H_2_O_2_, and that pretreatment with LIPUS decreased ROS accumulation induced by H_2_O_2_ (Figure 5A-C). Simultaneously, LIPUS suppressed H_2_O_2_-induced augmentation of MDA (Figure 5C), a biomarker of oxidative damage to lipids 31. Additionally, H_2_O_2_ upregulated the protein expression of 3-NT and 4-HNE, biomarkers of oxidative damage 32, which were downregulated by LIPUS (Figure 5D-F).

### LIPUS mitigates H_2_O_2_-induced oxidative stress via Nrf2 signaling pathway

**Figure 6:**
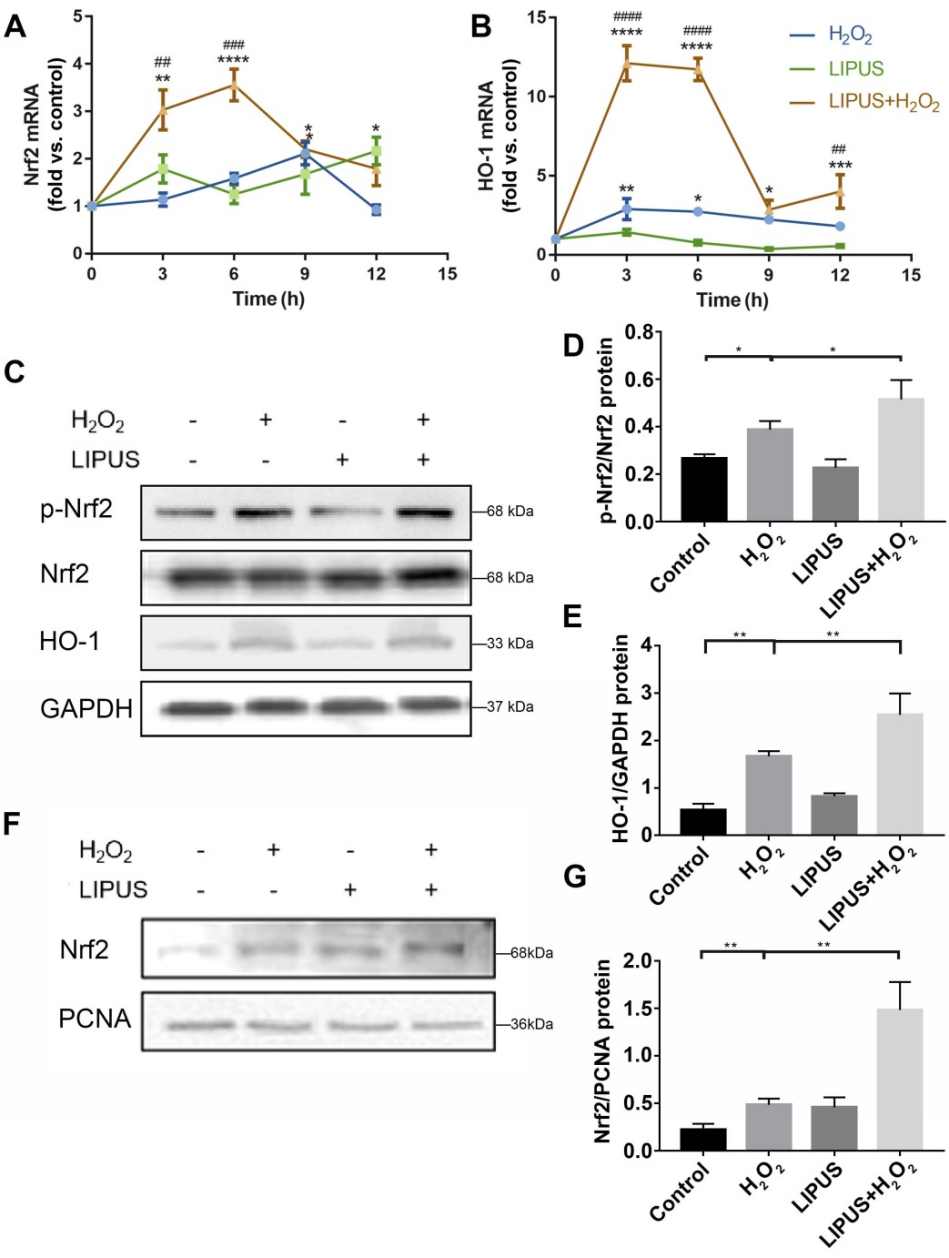
** LIPUS mitigates H_2_O_2_-induced oxidative stress via Nrf2 signaling pathway.** After H_2_O_2_-induced oxidative stress and LIPUS treatment, mRNA expression of (**A**) Nrf2 and (**B**) HO-1 was examined by qPCR. (**C**) Activation of phospho-Nrf2 and HO-1 were examined by western blot. The representative activation of (**D**) phospho-Nrf2 and (**E**) HO-1 were measured by semi-quantitative analysis. (**F**) Expression of Nrf2 in the nucleus was examined by western blot. (**G**) The quantitative expression of Nrf2 in the nucleus was measured by semi-quantitative analysis. Data are presented as the mean ± SEM (n = 3). *, *p* < 0.05; **, *p* < 0.005; ***, *p* < 0.0005; ****, *p* < 0.00005.

Nrf2 is known to play a critical role in resisting oxidative stress by transcriptional activation of anti-oxidative enzymes such as HO-1, which reduce ROS and alleviate cell damage 33. Nrf2 reportedly plays a pivotal role in oxidative responses 34. Therefore, whether LIPUS alleviates oxidative stress by activating the Nrf2 signaling pathway was examined. The expression levels of *Nrf2* and* HO-1* among different groups were remarkably distinct (Figure 6A-B). LIPUS, by itself, did not increase Nrf2 and HO-1 gene expression. H_2_O_2_ alone did not affect the genetic expression level of Nrf2 until 9 h after H_2_O_2_ exposure, whereas the level of HO-1 increased immediately after H_2_O_2_ exposure. LIPUS pretreatment significantly increased Nrf2 and HO-1 expression levels following exposure to H_2_O_2_, which remained high between 3 and 6 h. Therefore, the protein expression levels of Nrf2 and HO-1 were evaluated 6 h following exposure to H_2_O_2_. Western blotting results (Figure 6C-G) indicated that H_2_O_2_ exposure not only increased the activation of phospho-Nrf2 but also Nrf2 accumulation in the nucleus, which was further upregulated by LIPUS pretreatment. However, the expression of Nrf2 was not increased by exposure to H_2_O_2_.

### Knockdown of Nrf2 gene abrogates the protection of LIPUS against H_2_O_2_-induced oxidative stress

**Figure 7:**
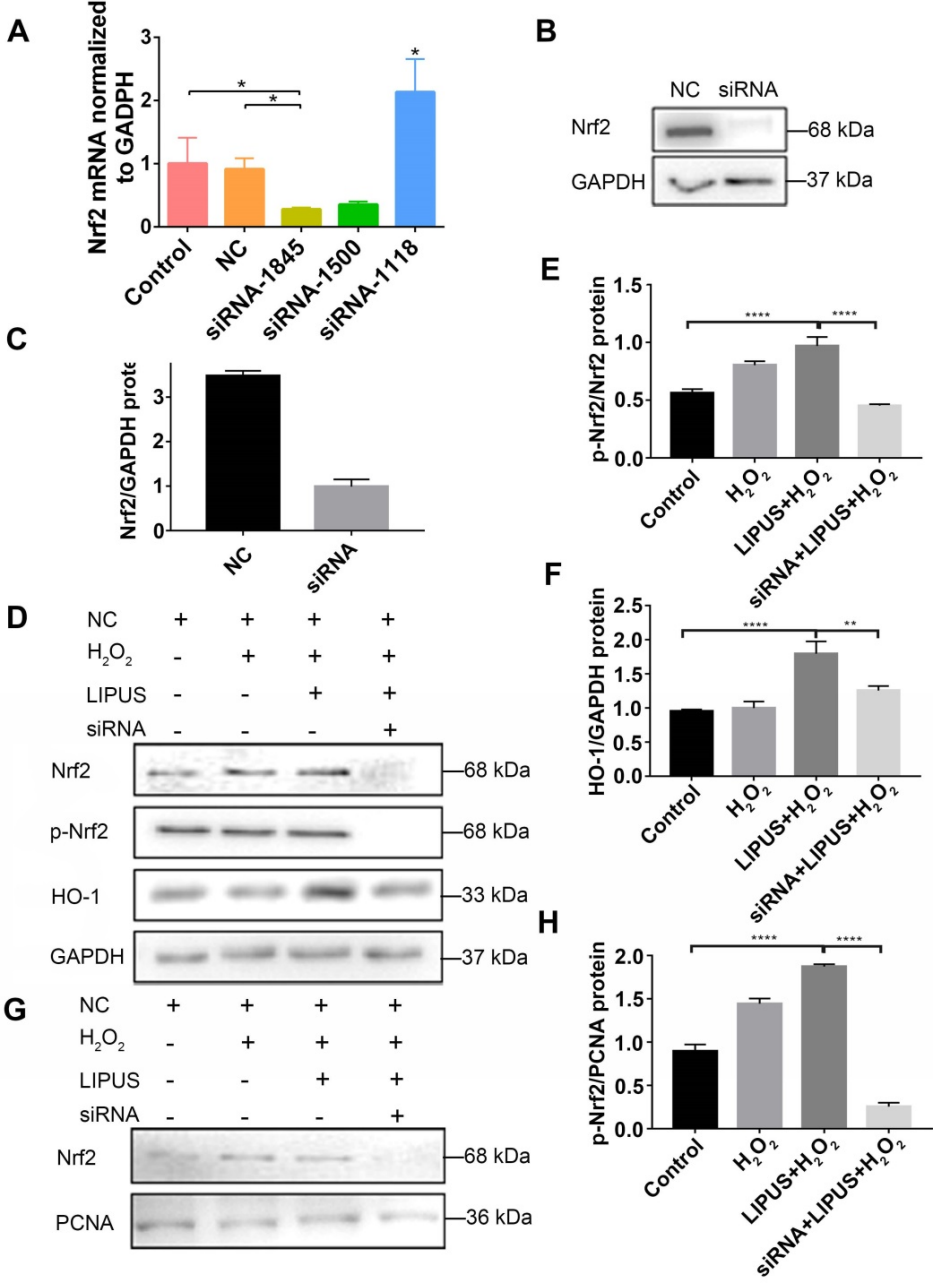
** Knockdown of Nrf2 gene abrogates the protection of LIPUS against H_2_O_2_-induced oxidative stress.** Transfection effects of small interfering RNA (siRNA) or negative control siRNA (NC) were analyzed by (**A**) qPCR and (**B**) western bloting. (**C**) The quantitative expression of Nrf2 in the nucleus was measured by semi-quantitative analysis. (**D**) After transfection with siRNA, activation of phospho-Nrf2 and HO-1 were examined by western blotting. The respective activation of (**E**) phospho-Nrf2 and (**F**) HO-1 were measured by semi-quantitative analysis. (**G**) Expression of Nrf2 in the nucleus was examined by western blot. (**H**) The quantitative expression of Nrf2 in the nucleus was measured by semi-quantitative analysis. Data are presented as the mean ± SEM (n = 3). *, *p* < 0.05; **, *p* < 0.005; ***,* p* < 0.0005; ****, *p* < 0.00005.

Application of siRNA knockdown was utilized to further determine distinct characteristics of Nrf2 that may be involved in the protection effect exerted by LIPUS on H_2_O_2_-induced oxidative stress. Transfection efficiency was confirmed by qPCR (Figure 7A) and western blotting (Figure 7B-C). Following exposure to H_2_O_2_, phospho-Nrf2 activation, HO-1 expression, and nuclear phospho-Nrf2 accumulation were upregulated compared with those of the negative control and further upregulated by LIPUS pretreatment. However, phospho-Nrf2 activation, HO-1 expression, and nuclear phospho-Nrf2 accumulation were decreased in the silenced group (Figure 7D-H). The above results indicate that LIPUS protects against H_2_O_2_-induced oxidative stress by activating the Nrf2 pathway.

### Knockout of *Nrf2* weakens the protective effect exerted by LIPUS on ligature-induced alveolar bone destruction in periodontitis

**Figure 8:**
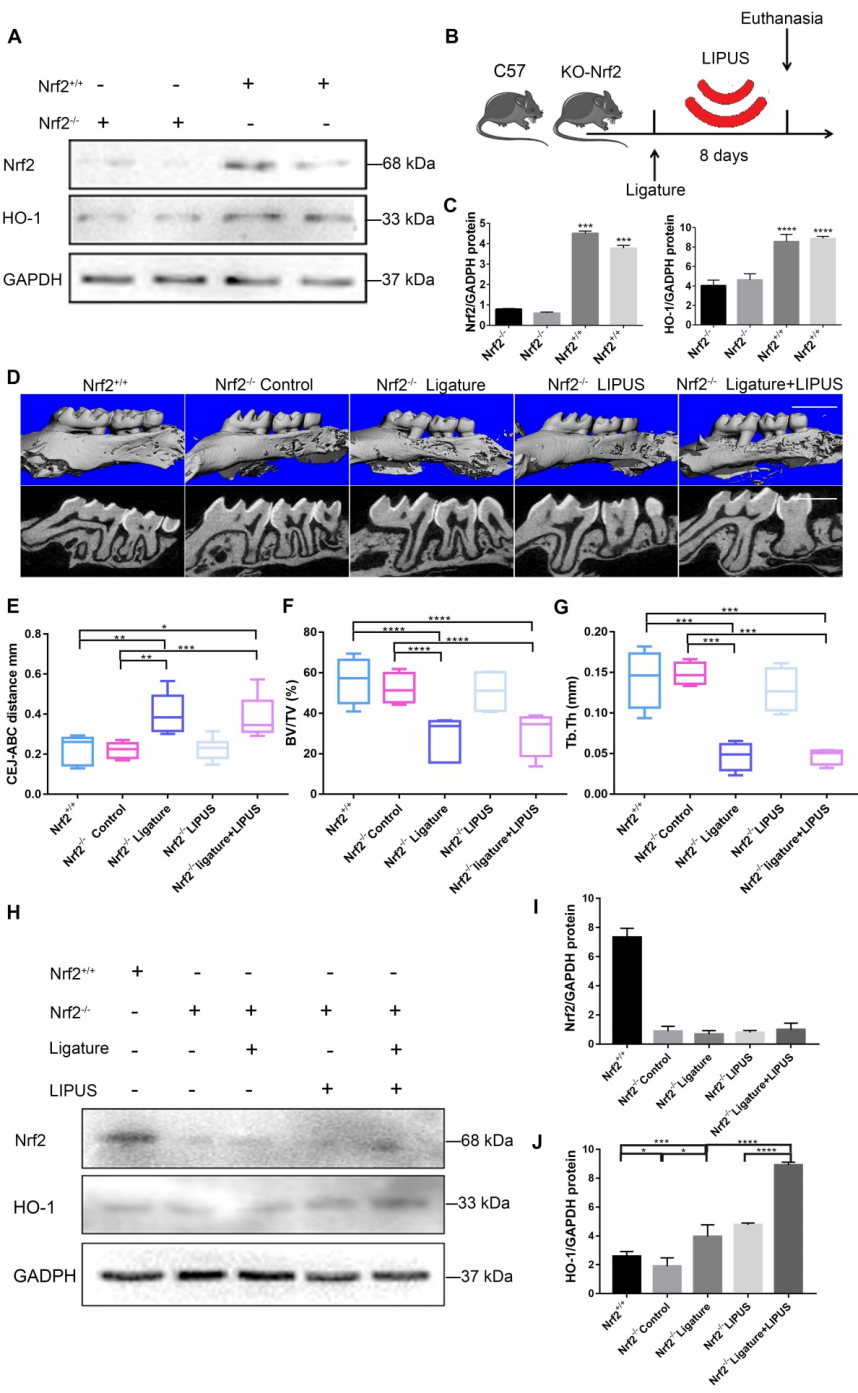
** Knockout of *Nrf2* weakens the protection effect exerted by LIPUS on ligature-induced alveolar bone destruction in periodontitis.** Nrf2^+/+^ mice and Nrf2^-/-^ mice were subjected to ligature insertion, with or without LIPUS treatment (30 min/day, with 200 ms pulses and 1.5 MHz) for 8 days simultaneously as described in Fig. 8B. Expression of Nrf2 and HO-1 was examined by western bloting. Activation of Nrf2 and HO-1 (**A**) quantitative levels (**C**). Three-dimensional image of the alveolar bone (Scale bar = 1 mm) (**D**). (**E**) CEJ-ABC distance, (**F**) BV/TV, and (**G**) Tb.Th. Nrf2 (**I**) and HO-1 (**J**) quantitative levels (**H**). Nrf2^+/+^ (WT with no treatment), Nrf2^-/-^ Control (Nrf2^-/-^ with no treatment), Nrf2^-/-^ Ligature (Nrf2^-/-^ with ligature-induced experimental periodontitis), Nrf2^-/-^ LIPUS (Nrf2^-/-^ with LIPUS treatment only), Nrf2^-/-^ Ligature+LIPUS (Nrf2^-/-^ with experimental periodontitis and LIPUS treatment). Data are presented as the mean ± SEM (n = 3). *, *p* < 0.05; **, *p* < 0.005; ***, *p* < 0.0005; ****, *p* < 0.00005.

**Figure 9:**
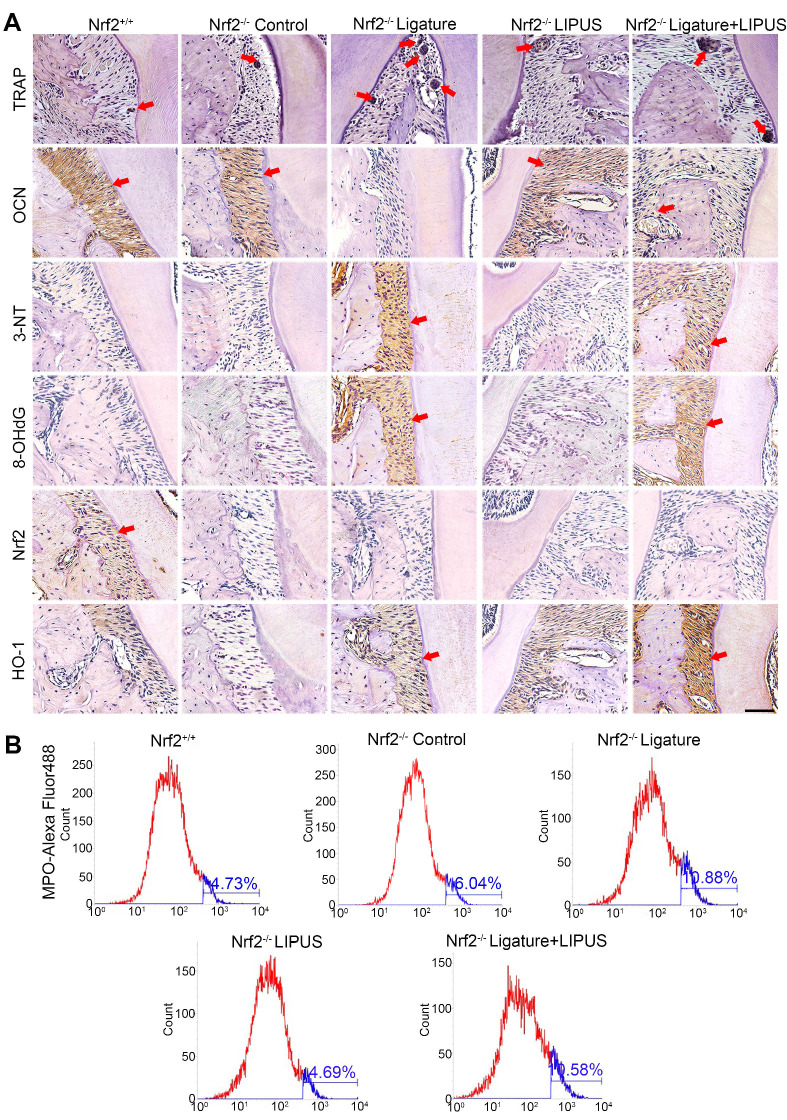
** Knockout of *Nrf2* abolishes the protection of LIPUS against ligature-induced oxidative stress.** (**A**) TRAP staining, immunohistochemical staining of OCN, 3-NT, 8-OHdG, Nrf2, and HO-1 at 400× magnification in periodontal tissue (Scale bar = 100 µm). (**B**) MPO flow analysis. Red arrows indicate positive staining.

In order to clarify the direct role of Nrf2 in LIPUS-mediated protection against periodontitis and bone resorption, ligature was applied to Nrf2^-/-^ mouse models to induce experimental periodontitis following which the mice were treated with LIPUS for 8 days simultaneously. Next, gingival tissues were harvested as illustrated (Figure 8B). A western blot revealed minimal Nrf2 or HO-1 expression in Nrf2^-/-^ mice (Figure 8A, C). These results indicated that deletion of *Nrf2* abolished the activation of Nrf2 and the expression of its antioxidant genes downstream. Compared with Nrf2^+/+^ mice, ligature-treated Nrf2^-/-^ mice showed a further decrease in bone resorption, where LIPUS did not rescue ligature-induced bone loss. Meanwhile, LIPUS-only treatment made no impact on destruction of alveolar bone (Figure 8D-G). Almost no Nrf2 expression was observed regardless of LIPUS treatment or experimental induction of periodontitis (Figure 8H-J). Interestingly, Nrf2 abrogation resulted in a moderate decrease of some antioxidants (HO-1). However, following LIPUS treatment, a significant upregulation of HO-1 was observed in Nrf2^-/-^ mice (Figure 8H-J). Hence, it is surmised that LIPUS treatment did not reverse alveolar bone destruction in Nrf2^-/-^ mice and that deletion of *Nrf2* may exacerbate HO-1 expression reactively. In addition, by the Nrf2^-/-^ ligature group, the osteogenesis markers ALP and OCN were decreased as compared with that in the Nrf2^+/+^ group and the Nrf2^-/-^ control group, and ALP and OCN showed insignificant increases in the Nrf2^-/-^ ligature + LIPUS group (Figure 9A and Figure S2). Significant induction of IL-6, TNF-α, RANKL, and TRAP staining was shown by histochemical detection in the Nrf2^-/-^ ligature groups, and LIPUS treatment did not decrease osteoclastogenesis in the Nrf2^-/-^ ligature + LIPUS groups (Figure 9A, Figure S2 and S3).

### Knockout of *Nrf2* abolishes the protection of LIPUS against ligature-induced oxidative stress

The oxidative damage markers, 3-NT and 8-OHdG, were evaluated to investigate the effect of Nrf2 on LIPUS-mediated protection against ligature-induced oxidative stress in experimental periodontitis. Immunohistochemistry indicated almost no Nrf2 expression with or without LIPUS treatment in Nrf2^-/-^ mouse experimental periodontitis models. Furthermore, significant HO-1 expression, observed in Nrf2^-/-^ mice with experimental periodontitis, was further boosted due to LIPUS treatment, compared with that of Nrf2^+/+^ mice (Figure 9A). Increased accumulation of 3-NT, 8-OHdG, and MPO was observed in Nrf2^-/-^ mice in which the ligature significantly induced oxidative damage, compared with that of the Nrf2^-/-^ control group and wild type. However, LIPUS treatment did not prevent these pathological changes in Nrf2^-/-^ mice. Hence, the deletion of *Nrf2* exacerbated ligature-induced oxidative damage and inhibited the protective effect of LIPUS against ligature-induced oxidative damage (Figure 9A and 9B).

### Inhibition of PI3K/Akt abrogated the protection of LIPUS against H_2_O_2_-induced oxidative stress

**Figure 10:**
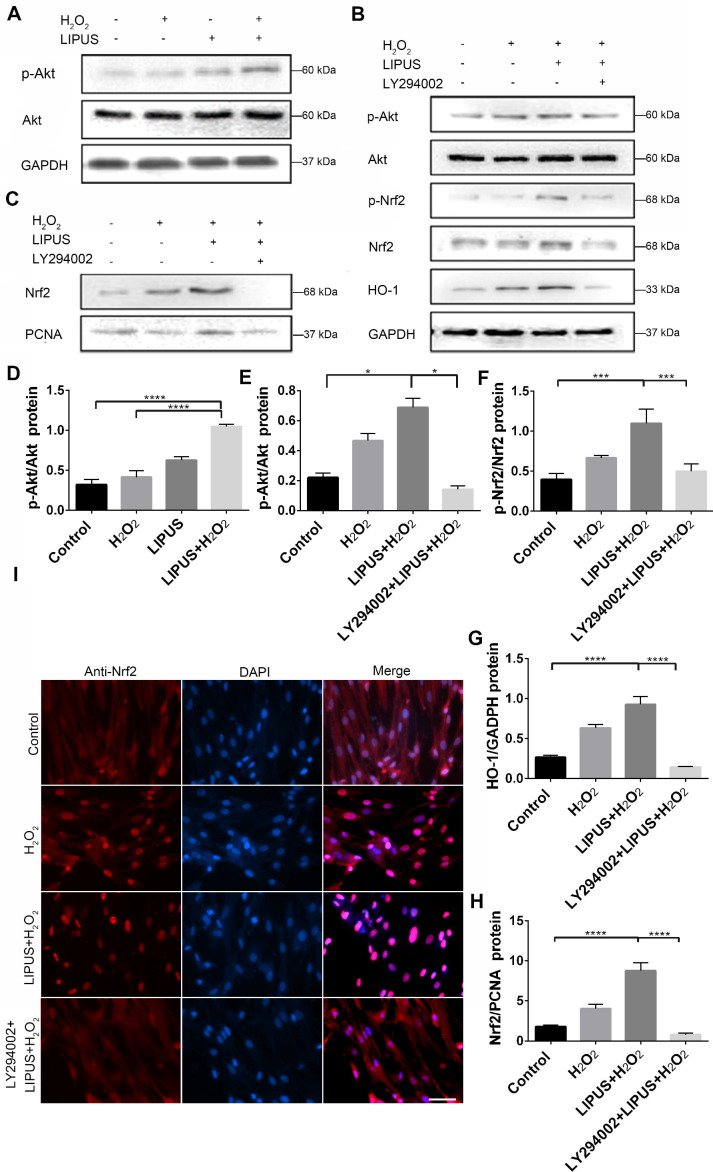
** Inhibition of PI3K/Akt abrogated the protection of LIPUS against H_2_O_2_-induced oxidative stress.** (**A**) Activation of phospho-Akt was examined by western blotting. (**D**) The quantitative activation of Nrf2 was measured by semi-quantitative analysis. (**B**) Pretreatment of LY294002 (10 µM), activation of phospho-Akt, phospho-Nrf2 and HO-1 were examined by western blotting. The respective activations of phospho-Akt (**E**), phospho-Nrf2 (**F**) and HO-1 (**G**) were measured by semi-quantitative analysis. (**C**) Expression of Nrf2 in the nucleus was examined by western blot. (**H**) The quantitative expression of Nrf2 in the nucleus was measured by semi-quantitative analysis. (**I**) Nrf2 translocation was determined by immunofluorescence at 200× magnification (Scale bar = 200 µm). Red: Nrf2-staining, blue: nucleus (DAPI), and pink: merger of blue and red indicating nuclear localization of Nrf2. Data are presented as the mean ± SEM (n = 3). *, *p* < 0.05; **, *p* < 0.005; ***, *p* < 0.0005; ****, *p* < 0.00005.

PI3K/Akt is a well-known upstream regulator of Nrf2 35. LY294002, an inhibitor of PI3K/Akt 35, was used to further confirm whether LIPUS acts against H_2_O_2_-induced oxidative stress via the PI3K/Akt pathway. Western blotting indicated that LIPUS pretreatment significantly increased the activation of phospho-Akt following H_2_O_2_ exposure (Figure 10A, D). However, LY294002 reversed the LIPUS-promoted activation of phospho-Akt and phospho-Nrf2, blocked the LIPUS-facilitated expression of HO-1, and inhibited the LIPUS-mediated upregulation of nuclear Nrf2 (Figure 10B-C, E-H). Immunofluorescence analysis using anti-Nrf2 further confirmed these results by indicating that H_2_O_2_ enhanced Nrf2 accumulation in the nucleus, where LIPUS pretreatment further accelerated it. However, LY294002 reversed its translocation into the nucleus (Figure 10I). These results indicate that LIPUS exerted a protective effect against H_2_O_2-_induced oxidative stress via the PI3K-Akt/Nrf2 pathway.

## Discussion

Periodontitis is among the most widespread inflammatory diseases 1. Multiple studies exploring the modalities in periodontitis treatment are being conducted 36. Conventional efforts have focused on pathogen elimination 37, inflammation control 37, and immunity modulation 38. However, current treatments have been found to be unsatisfactory, and the need for novel treatment options has been recognized. The current study applied LIPUS to manage alveolar bone homeostasis in periodontitis. Moreover, it was realized that reducing oxidative stress may provide a new perspective for future studies associated with periodontal treatment.

LIPUS is a clinically established, Food and Drug Administration (FDA)-approved therapy that is widely used to enhance bone growth during healing of non-union fractures and other osseous defects 39-41. LIPUS induces mechanical stress stimulating osteogenesis via modulation of calcium ion channels 42, 43. Previously, Li *et al*. first revealed that LIPUS may prevent bone loss due to osteoporosis 43. Later, Yue *et al*. demonstrated that LIPUS enhanced osteogenesis of adipose stem cells by upregulating osteogenic genes 44. Meanwhile, Carina *et al*. and Meng *et al*. implied that LIPUS treatment effectively impaired osteoclast differentiation and produced a reduction in osteoclast markers. Most importantly, our previous studies have shown that LIPUS exerts potential protective effects on alveolar bone regeneration in periodontal injury models 11 and facilitates osteogenic differentiation of PDLCs 12. These results were confirmed by micro-CT imaging, CEJ-ABC distances, BV/TV, Tb.Th., and H&E staining, which revealed that LIPUS significantly attenuated ligature-induced alveolar bone loss (Figure 1). Histology staining also showed that LIPUS prevented alveolar bone loss, which was associated with increased osteoblast differentiation and low osteoclast activity. The above results clearly indicate that LIPUS exerts a protective effect on alveolar bone destruction in ligature-induced periodontitis. Also, many case-control studies have reported a direct link between periodontitis and oxidative stress 19, 45-47. However, evidence indicating that oxidative stress plays a direct role in LIPUS protection is lacking. The findings of the current study led to the hypothesis that LIPUS moderate alveolar bone destruction in ligature-induced periodontitis via oxidative stress.

Oxidative stress is a redox state leading to excessive generation of ROS that causes severe damage to cells and tissues 48-50. Periodontitis is a widespread oral disease which is associated with high ROS levels 51. In previous clinical studies, high levels of ROS have been found to be elevated in the periodontal tissue of patients with periodontitis 52. Heightened oxidative stress in periodontal tissues results in the release of large amounts of ROS 53. Increased intracellular ROS levels were shown to support osteoclast differentiation and subsequent bone resorption 54. Meanwhile, the oxidative stress-induced lower activity of antioxidant enzymes suppressed osteogenic differentiation and aggravated the pathological process of periodontitis 19. Additionally, proteins and genes are major targets of oxidative damage 55. 3-NT represents a commonly used biomarker for protein oxidation 56, and 8-OHdG represents the most widely accepted assay for DNA oxidative damage 57. The present study confirms that ligatures induced oxidative damage by significantly increasing 3-NT and 8-OHdG in our experiment periodontal models, both of which were reversed by LIPUS. These results implied that LIPUS mitigated alveolar bone resorption in periodontitis by downregulating oxidative stress.

These observations prompted us to consider whether LIPUS facilitates osteogenic differentiation in PDLCs by reducing oxidative stress. H_2_O_2_ acts similarly to ROS, which permeate through cell membranes to initiate oxidative stress 58. Numerous reports have indicated that H_2_O_2_ may be used to establish an *in vitro* oxidative stress model 59-61. As indicated by the current study, following exposure to 200 µM H_2_O_2_ for 6 h, PDLCs displayed inhibited cell viability, suppressed osteogenic differentiation, and promoted osteoclast activity. However, LIPUS exerted cytoprotective activity by recovering cell viability and promoting osteogenic differentiation in H_2_O_2_-exposed cells. Intracellular ROS has been reported to be an indicator that directly reflects oxidative stress levels 61-63. Oxidative stress is a consequence of an increase in oxidizing species or a decrease in antioxidant levels 64. The extent of oxidative stress may be indicated by MDA as well. The present study demonstrates that the levels of ROS and MDA were significantly increased following H_2_O_2_ exposure but were reversed by LIPUS treatment. Additionally, oxidative stress also led to lipid peroxidation and resulted in 4-HNE production 65. The present results show that the high expression levels of 3-NT and 4-HNE caused by H_2_O_2_ exposure were overturned by LIPUS in PDLCs. Therefore, these results reveal that LIPUS significantly attenuated H_2_O_2_-induced oxidative stress, inhibition of cell viability, and suppression of osteogenic differentiation. Considered together, the results of the present study indicate that LIPUS alleviates osteogenesis differentiation in periodontal tissue by downregulating oxidative stress.

Nrf2, a master regulator of cellular redox homeostasis, activates cellular defense against oxidative stress by inducing hundreds of antioxidant and detoxifying enzymes 66, 67. Cytoplasmic Nrf2, which is released from the Keap1-Nrf2 complex in response to oxidative stress, binds to antioxidant response elements in the promoter region of many genes encoding antioxidant enzymes 67. Upregulation of antioxidant enzymes, most of which are regulated by Nrf2, has been shown to inhibit oxidative stress 68. The expression of Nrf2 and the downstream antioxidant enzyme, HO-1, were increased in ligature-induced alveolar bone destruction. Besides, Nrf2 expression and its function were further upregulated following LIPUS treatment. Based on these findings, we surmised that LIPUS-associated protection may be strongly linked to Nrf2-mediated antioxidant defense mechanisms. Following its detachment from the Keap1-Nrf2 complex due to anti-oxidative stress, phospho-Nrf2 is activated and translocated to the nucleus and causes induction of antioxidant gene transcription 69, 70. Therefore, western blot and qPCR were utilized to examine the activation of phospho-Nrf2, its nuclear translocation, and transcription of cytoprotective genes. The results indicate that LIPUS activated phospho-Nrf2, increased Nrf2 in the nucleus, and upregulated HO-1 expression. Previous studies have revealed that disruption of Nrf2 leads to increased oxidative stress and suppression of antioxidant capacity 71. Furthermore, silencing Nrf2 expression significantly suppressed antioxidant capacity in PDLCs regardless of LIPUS treatment. LIPUS did not significantly alleviate ligature-induced promotion of osteogenic differentiation and suppression of osteoblast activity, which leads to an increase in bone resorption in Nrf2-deficient mice. LIPUS had no effects on ligature-induced oxidative damage in Nrf2^-/-^ mouse models. These results clarify the pivotal role played by Nrf2-mediated antioxidant defense mechanisms *in vitro* and *in vivo*, and indicated that LIPUS alleviates oxidative stress and alveolar bone resorption via Nrf2 activation in periodontitis.

Upregulation of Nrf2 in PDLCs by LIPUS required further clarification. PI3K/Akt is a well-known upstream regulator of Nrf2 and activation of PI3K/AKT directly decreases Nrf2 degradation by promoting Nrf2 phosphorylation and accelerating its translocation into nuclei 26, 72. Decreased Nrf2 degradation may initiate the transcription of downstream antioxidant genes that defend against oxidative stress 71. Coincidentally, several past studies have indicated that LIPUS activated the PI3K/AKT pathway, thereby promoting its function 26, 73. In order to elucidate the mechanism underlying the above process, we examined whether PI3K/Akt is mediated by LIPUS via upregulation of Nrf2 expression. Western blot and immunofluorescence showed that LIPUS upregulated the activation of phospho-Nrf2 and its accumulation in the nucleus, in an oxidative stress micro-environment. To investigate the role of the PI3K/Akt pathway further, LY294002, a pharmacological inhibitor of the PI3K/Akt pathway, was used. Inhibition of the PI3K/Akt signaling pathway markedly blocked Nrf2 and its downstream antioxidant expression, which confirmed that the PI3K-AKT pathway facilitates the regulation of Nrf2 by LIPUS under oxidative stress. These results suggest that LIPUS alleviated alveolar bone destruction in periodontitis by diminishing oxidative stress, and that this process was partially facilitated by PI3K-Akt/Nrf2 signaling.

## Conclusions

In conclusion, the ligature technique was used to produce an experimental periodontitis model that mimicked conditions of enhanced oxidative stress. To the best of our knowledge, the current study is the first to provide evidence indicating that LIPUS alleviates suppressed osteogenic differentiation and alveolar bone destruction due to periodontitis, by reducing oxidative stress. The Nrf2-mediated antioxidant defense mechanism, which plays an essential role in the protective effect against alveolar bone destruction, was upregulated and activated by LIPUS, and the PI3K/Akt-Nrf2 pathway played a partial role in this process. From a clinical point of view, these findings indicated that future studies of periodontal treatment modalities should pay more attention to the management of oxidative stress. LIPUS could be used as a physical therapy to promote periodontal regeneration after conventional treatment such as debridment and surface planning in periodontitis and diabetic periodontitis since oxidative stress was higher in diabetic periodontitis.

## Supplementary Material

Supplementary figures and tables.

## Abbreviations

3-NT: 3-nitrotyrosine
4-HNE: 4-Hydroxynonenal
8-OHdG: 8-Hydroxy-2'-deoxyguanosine
ALP: alkaline phosphatase
BV/TV: bone volume over total volume
CCK-8: cell Counting Kit-8
CEJ-ABC: cemento-enamel junction and alveolar bone crest
DAB: Diaminobenzidine
EDTA: ethylene diamine tetraacetic acid
FBS: fetal bovine serum
GAPDH: glyceraldehyde-3-phosphate dehydrogenase
HO-1: heme oxygenase-1
IL-6: interleukin-6
LIPUS: low intensity pulsed ultrasound
Micro-CT: Micro-computed tomography
MPO: myeloperoxidase
Nrf2: nuclear factor erythroid 2-related factor 2
OCN: osteocalcin
PBS: phosphate-buffered saline
PDLCs: periodontal ligament cells
PMSF: phenylmethanesulfonyl fluoride
RANKL: receptor activator for nuclear factor- κ B ligand
ROS: reactive oxygen species
Runx2: runt-related transcription factor-2
SD: Sprague-Dawley
SDS-PAGE: sodium dodecyl sulfate-polyacrylamide gel electrophoresis
siRNA-NC: siRNA-Negative control
Th.Tb: trabecular thickness
TNF-α: tumor necrosis factor-alpha
TRAP: tartrate-resistant acidic phosphatase
