# Low-intensity Pulsed Ultrasound regulates alveolar bone homeostasis in experimental Periodontitis by diminishing Oxidative Stress: Erratum

**DOI:** 10.7150/thno.69529

**Published:** 2022-01-01

**Authors:** Siqi Ying, Minmin Tan, Ge Feng, Yunchun Kuang, Duanjing Chen, Jie Li, Jinlin Song

**Affiliations:** 1 College of Stomatology, Chongqing Medical University, Chongqing, China.; 2 Chongqing Key Laboratory of Oral Diseases and Biomedical Sciences, Chongqing, China.; 3 Chongqing Municipal Key Laboratory of Oral Biomedical Engineering of Higher Education, Chongqing, China.

The authors regret that the image wrongly attached due to their carelessness in integrating figures in the Figure 2A, Figure 9A and Supplement figure 1. The correct versions are shown below. These images substitution would not affect any results presented in the originally-published version, nor the corresponding text description and the conclusion of the paper. The authors apologize for any inconvenience or misunderstanding that this error may have caused.

## Figures and Tables

**Figure 2:**
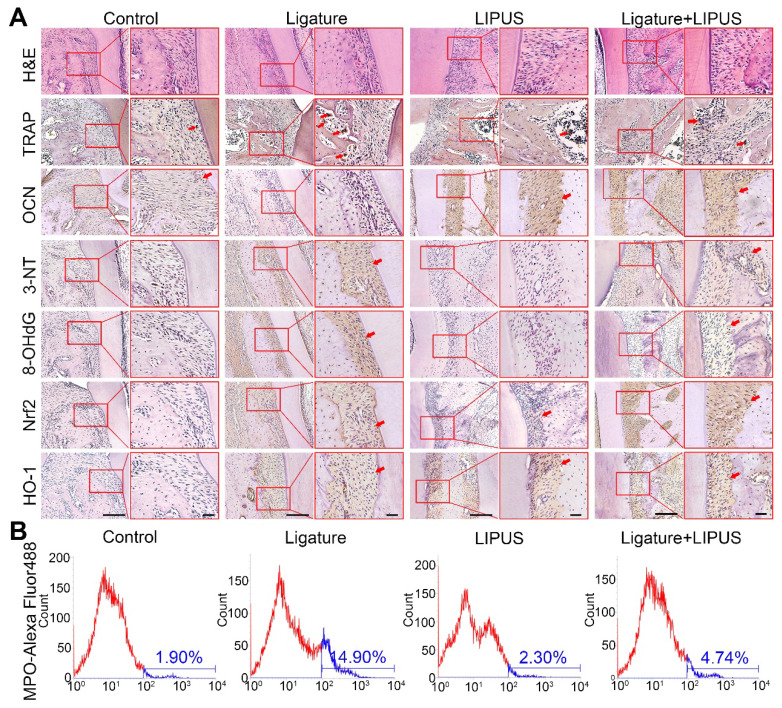
** LIPUS alleviates ligature-induced oxidative stress via Nrf2 Pathway.** (**A**) H&E staining at 200× and 400× magnification. TRAP staining, immunohistochemical staining of OCN, 3-NT, 8-OHdG, Nrf2, HO-1 at 200× and 400× (red box) magnification (Scale bar = 100 µm). (**B**) MPO flow analysis. Red arrows indicate positive staining.

**Figure 9:**
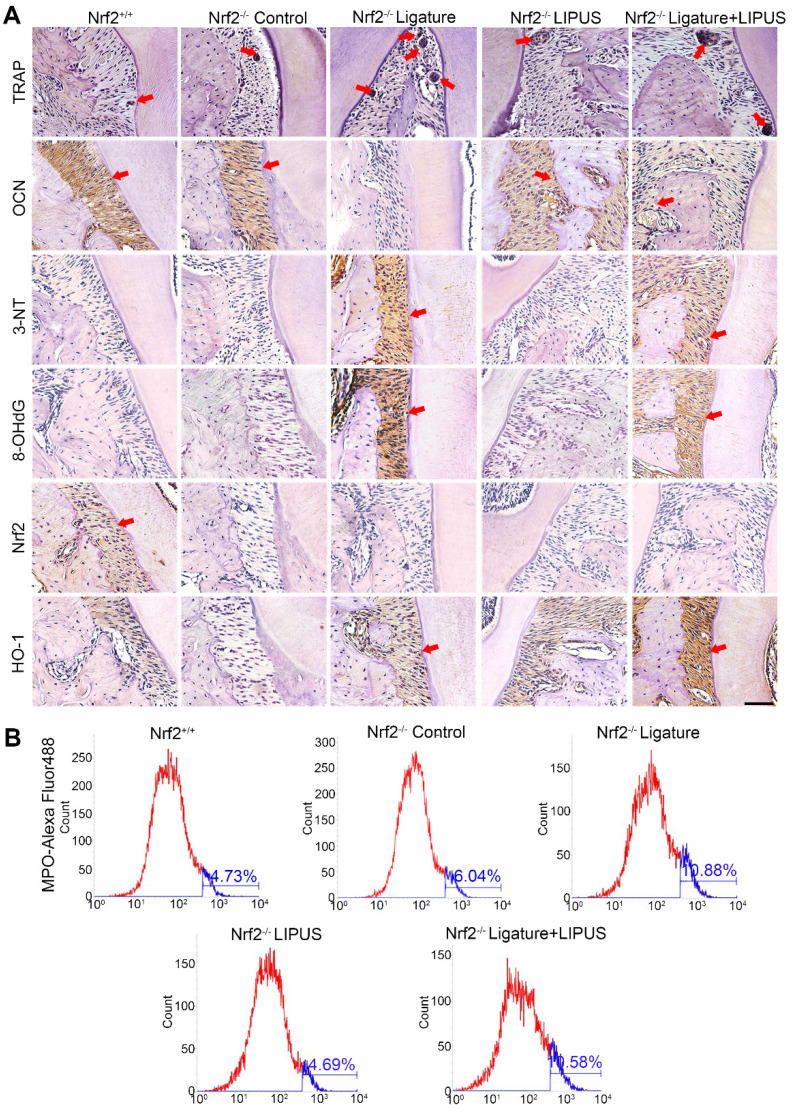
** Knockout of *Nrf2* abolishes the protection of LIPUS against ligature-induced oxidative stress.** (**A**) TRAP staining, immunohistochemical staining of OCN, 3-NT, 8-OHdG, Nrf2, HO-1 at 400× magnification in periodontal tissue (Scale bar = 100 µm). (**B**) MPO flow analysis. Red arrows indicate positive staining.

**Figure A:**
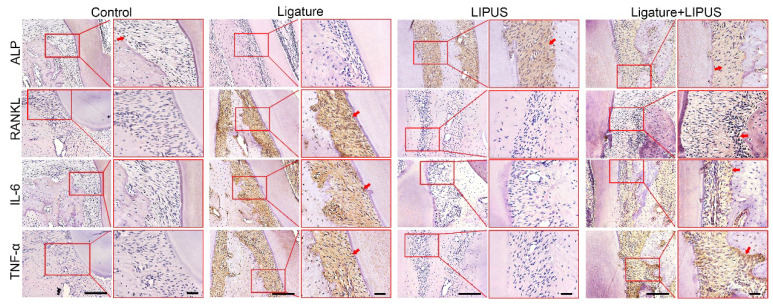
** Supplementary** **Figure 1.** Immunohistochemical staining of ALP, RANKL, IL-6, TNF-α at 200× and 400× magnification in rats (Scale bar = 100 µm). Red arrows indicate positive staining.

