# Supplementary material for: Low-intensity Pulsed Ultrasound regulates alveolar bone homeostasis in experimental Periodontitis by diminishing Oxidative Stress
Source: Theranostics. 2020 Aug 1;10(21):9789–807. doi: 10.7150/thno.42508 (PMC7449900; doi:10.7150/thno.42508)
Supplement: Supplementary file 1 — Supplementary figures and tables. [file thnov10p9789s1.pdf]

Supplementary Material

Supplementary figures.

Supplementary Figure 1. Immunohistochemical staining of ALP, RANKL, IL-6, TNF-α at 200× and 400× magnification in rats (Scale bar = 100 μm). Red arrows indicate positive staining.

Supplementary Figure 2. Immunohistochemical staining of ALP, RANKL, IL-6, TNF-α at 400× magnification in periodontal tissue (Scale bar = 100 μm). (B) MPO flow analysis. Red arrows indicate positive staining.

Supplementary Figure 3. The semi-quantitative analysis of osteogenic (A), oxidative stress (B) and osteoclastogenic (C) markers by immunohistochemical staining in rats. (D) Osteoclasts number by TRAP staining in rats. Control (no treatment), Ligature (ligature-induced experimental periodontitis), LIPUS (only LIPUS treatment), and Ligature + LIPUS (experimental periodontitis before LIPUS treatment). The semi-quantitative analysis of osteogenic (E), oxidative stress (F) and osteoclastogenic (G) markers by immunohistochemical staining in mice. (H) Osteoclasts number by TRAP staining in mice. $Nrf2^{+/+}$ (WT with no treatment), $Nrf2^{-/-}$ Control ($Nrf2^{-/-}$ with no treatment), $Nrf2^{-/-}$ Ligature ($Nrf2^{-/-}$ with ligature-induced experimental periodontitis), $Nrf2^{-/-}$ LIPUS ($Nrf2^{-/-}$ with LIPUS treatment only), $Nrf2^{-/-}$ Ligature + LIPUS ($Nrf2^{-/-}$ with experimental periodontitis and LIPUS treatment). Data are presented as the mean ± SEM (n = 3). *, $p < 0.05$; **, $p < 0.005$; ***, $p < 0.0005$; ****, $p < 0.00005$.

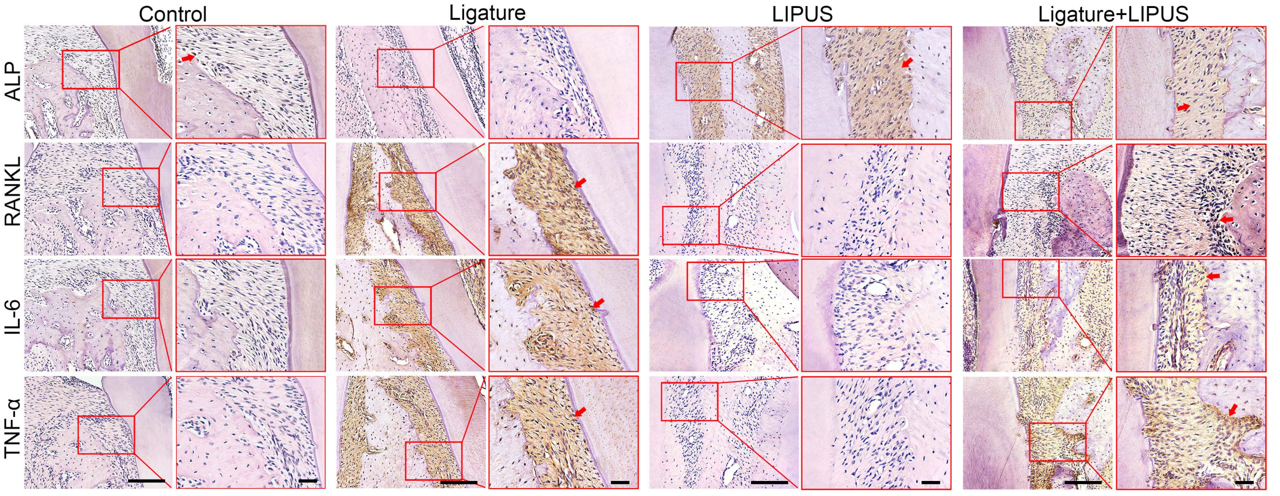

Control
Ligature
LIPUS
Ligature+LIPUS
ALP
RANKL
IL-6
TNF-α

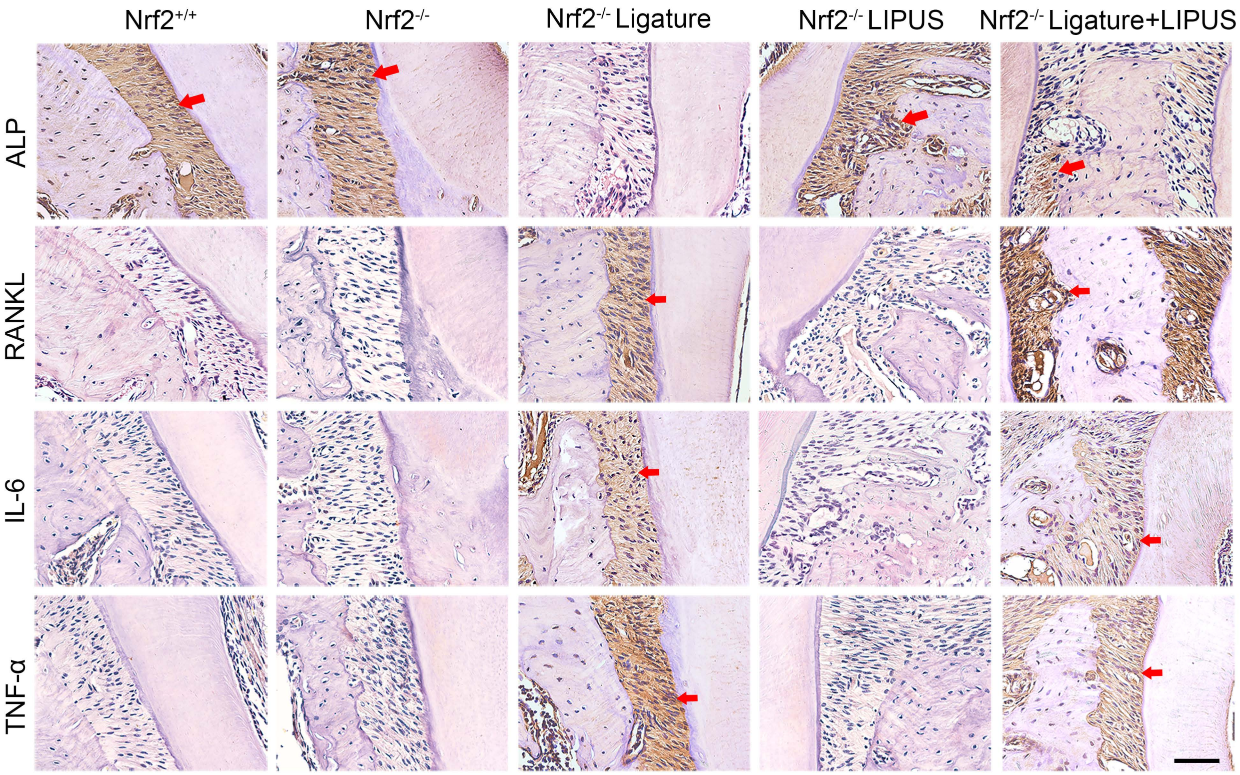

Nrf2+/+
Nrf2-/-
Nrf2-/- Ligature
Nrf2-/- LIPUS
Nrf2-/- Ligature+LIPUS
ALP
RANKL
IL-6
TNF-α

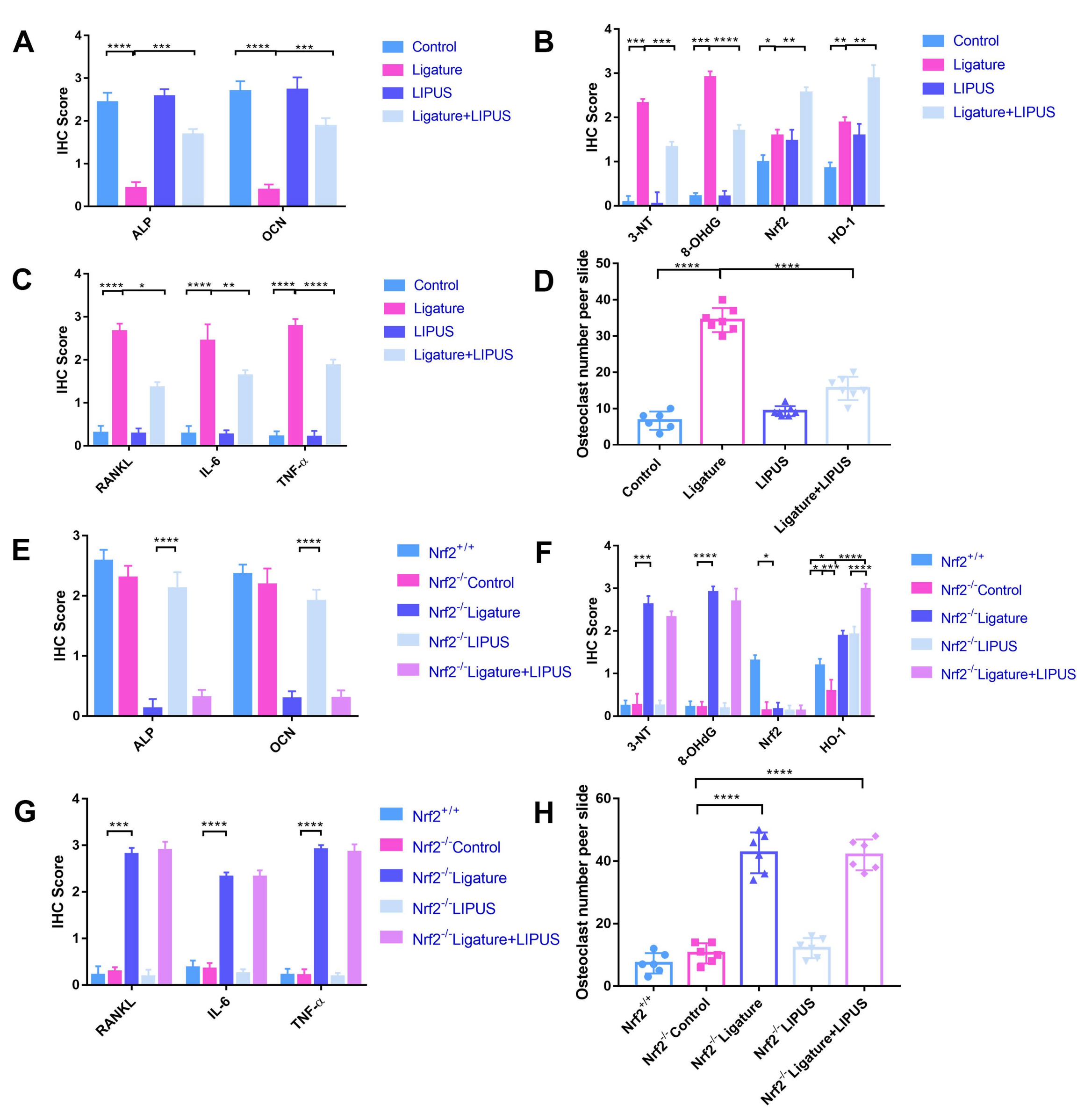
A
IHC Score
ALP
OCN
Control
Ligature
LIPUS
Ligature+LIPUS
B
3-NT
8-OHdG
Nrf2
HO-1
C
RANKL
IL-6
TNF-α
D
Osteoclast number peer slide
E
Nrf2+/+
Nrf2-/-Control
Nrf2-/-Ligature
Nrf2-/-LIPUS
Nrf2-/-Ligature+LIPUS
F
G
H
